# Uncommon Ophthalmic Artery Involvement in Spontaneous Vertebral Artery Dissection in a Young Woman With Optic Neuritis: A Case Report and Literature Review

**DOI:** 10.1155/crvm/8169088

**Published:** 2026-04-08

**Authors:** Jaime Said, Andrew Rhim, Dennis Cardriche, Thomas H. Matese

**Affiliations:** ^1^ Emergency Medicine Department, Emergency Medicine Residency Program, HCA Florida St. Lucie Hospital, Port Saint Lucie, Florida, USA

**Keywords:** cervical artery dissection, ischemic optic neuropathy, ophthalmic artery stenosis, optic neuritis, vertebral artery dissection, young adult stroke

## Abstract

We report a 36‐year‐old healthy woman with 2 days of right‐sided blurry vision, headache, and pain with eye movement. Exam showed decreased visual acuity, focal retinal pallor, and abnormal cranial nerve II findings. CTA revealed right vertebral artery dissection at C5–C6 with concurrent ophthalmic artery narrowing. She received anticoagulation, intravenous steroids for optic neuritis, and improved with follow‐up. This rare presentation underscores the need to consider cervical artery dissection in young patients with painful vision loss and subtle fundoscopic changes, as timely vascular imaging can reveal unexpected concurrent pathology.

## 1. Introduction

Vertebral artery dissection (VAD) is a rare etiology of ischemic stroke in younger adults under the age of 50 [1, 2]. Arterial dissection accounts for approximately 4%–5% of all ischemic strokes and is responsible for up to 25% of strokes occurring in young‐ and middle‐aged adults [2, 3]. Although traumatic causes are well known, VAD can also occur spontaneously or in association with connective tissue disorders [1, 4, 5]. It most commonly involves segment two and three of the vertebral artery, particularly at the C1–C2 level, and is a frequent contributor to posterior circulation ischemia [1, 4]. Clinical symptoms of VAD can include headache, neck pain, dizziness, dysarthria, ataxia, and visual disturbances, though the presentation can be subtle or atypical [5, 6]. Computed tomography angiography (CTA) and magnetic resonance angiography (MRA) are the primary imaging modalities used to diagnose VAD and ophthalmic artery stenosis [7]. The standard treatment options for vertebral artery dissection, as recommended by the American College of Cardiology and associated societies, include antithrombotic therapy and, in select cases, surgical or endovascular revascularization [8]. Guidelines suggest that antithrombotic treatment with either an anticoagulant (e.g., heparin, low molecular weight heparin, and warfarin) or a platelet inhibitor (e.g., aspirin, clopidogrel, or the combination of extended‐release dipyridamole plus aspirin) for at least 3–6 months is reasonable for patients with extracranial vertebral arterial dissection associated with ischemic stroke or transient ischemic attack (TIA) [8]. Carotid angioplasty and stenting may be considered when ischemic symptoms do not respond to medical management [8].

Ophthalmic artery involvement in the context of VAD is extremely rare, given that the ophthalmic artery arises from the internal carotid artery and is not part of the vertebrobasilar circulation [9, 10]. Ophthalmic artery stenosis is a serious condition that can result in acute visual impairment due to compromised retinal or optic nerve perfusion. Clinical symptoms of ophthalmic artery stenosis typically include acute, painless monocular vision loss, which may be transient (amaurosis fugax) or persistent [11]. Additional findings may include optic disc edema if perfusion to the optic nerve head is compromised, and in some cases, visual field defects depending on the extent of retinal ischemia [11]. A comprehensive systemic evaluation is essential to identify potential underlying etiologies, including embolic, inflammatory, or infectious processes. This workup should include a detailed medical history, thorough physical examination, and laboratory testing—such as erythrocyte sedimentation rate (ESR) and C‐reactive protein (CRP)—to rule out conditions like giant cell arteritis (GCA), which may present similarly [12]. Treatment strategies are tailored to the underlying causes. In cases of inflammatory or autoimmune origin, high‐dose corticosteroids are the mainstay of therapy [11, 12]. For embolic or thrombotic causes, antiplatelet or anticoagulant therapy may be initiated, depending on the clinical scenario and patient‐specific risk factors [13]. Early recognition and intervention are critical to preserving vision and preventing further vascular compromise.

In this report, we present a rare case of spontaneous right VAD at the C5–C6 level with concurrent narrowing of the right ophthalmic artery in a previously healthy 36‐year‐old woman, who presented with right‐sided blurry vision and headache. She was initially managed with anticoagulation and transferred to a tertiary care center for specialized ophthalmologic and vascular surgery evaluation. Further workup revealed right optic neuritis in the absence of a demyelinating disease process, confirmed by magnetic resonance imaging (MRI) and lumbar puncture. The patient responded well to intravenous corticosteroids and was discharged in stable condition with close outpatient follow‐up.

## 2. Case Presentation

A 36‐year‐old previously healthy woman presented to the emergency department with right‐sided blurry vision and a dull, persistent headache of 2 days′ duration. She denied trauma, recent neck manipulation, or any identifiable precipitating events. She also reported pain with right eye movement. There were no associated neurological deficits such as vertigo, limb weakness, dysphagia, or ataxia.

Her vitals on arrival included a blood pressure of 154/105 mmHg, heart rate of 72 beats/min, respiratory rate of 18/min, temperature of 36.7°C, and O2 saturation of 100% on room air. Initial ophthalmologic examination revealed a notable decrease in visual acuity in the right eye: 20/200(R) and 20/20(L). The fundoscopic exam showed right upper retinal paleness at the 4 o′clock position. Ocular tonometry revealed right eye 22 mmHg and left eye 21 mmHg (normal range: 15–25 mmHg). The neurologic exam was unremarkable apart from an abnormal cranial nerve II finding (changes in visual acuity). Initial laboratory values are seen in Table 1.

**Table 1:** Laboratory values on initial emergency room presentation.

	Patient values	Reference values		Patient values	Reference values
**Hematology**			**Chemistry**		
White blood cells (WBC)	6.2	4.0–10.5 10^3^/u	Sodium	139	135–145 mmol/L
Red blood cells (RBC)	4.52	3.93–5.22 10^6^/uL	Potassium	3.4 L	3.5–5.2 mmol/L
Hemoglobin	12.2	11.2–15.7 G/DL	Chloride	108	95–110 mmol/L
Hematocrit (%)	37.3	34.1%–44.9%	Carbon dioxide	26	19–34 mmol/L
Mean corpuscular volume	82.5	79.4–94.8 fL	BUN	10	6–22 mg/dL
Mean corpuscular hemoglobin	27	25.6–32.2 pg	Creatinine	0.69	0.43–1.13 mg/dL
Mean corpuscular hemoglobin concentration	32.7	32.2–35.5 g/dL	Est GFR	> 90	>/=90
Red cell distribution width (%)	15	11.7%–14.4%	Glucose	78	70–110 mg/dL
Platelet count	261	150–400 10^3^/uL	Calcium	9.2	8.4–10.2 mg/dL
Mean platelet volume	11.5	9.4–12.3 fL	C‐Reactive Protein	0.6	0–1.0 mg/dL
Nucleated RBC (%)	0	0.0%–0.2%			
Immature granulocytes (%)	0.6	0.0%–0.4%	**Coagulation**		
Immature granulocytes (count)	0.04	0.00–0.03 10^3^/uL	APTT	35	25–38 s
Neutrophils (count)	3.71	1.70–2.80 10^3^/uL			
Segmented neutrophils (%)	59.6	34.0%–71.1%			
Lymphocytes (%)	31.8	19.3%–51.7%			
Lymphocytes (count)	1.98	1.18–3.74 10^3^/uL			
Monocytes (%)	5.9	4.7%–12.5%			
Monocytes (count)	0.37	0.24–0.63 10^3^/uL			
Eosinophils (%)	1.8	0.7–5.8%			
Eosinophils (count)	0.11	0.04–0.36 10^3^/uL			
Basophils (%)	0.3	0.1%–1.2%			
Basophils (count)	0.02	0.01–0.08 10^3^/uL			
Nucleated RBCs (count)	0	0.00–0.18 10^3^/uL			
Erythrocyte sedimentation rate	38	0–20 mm/hr			

Noncontrast CT of the head was unremarkable. CTA of the head and neck revealed a right VAD at the C5–C6 level and narrowing of the right ophthalmic artery (Figures 1a, 1b, and 1c). The patient was placed on intravenous heparin for anticoagulation and transferred to a tertiary care center with access to ophthalmology and vascular surgery consultations. MRI of the orbits revealed findings consistent with right optic neuritis (Figure 2a, b). She received high‐dose intravenous methylprednisolone for 3 days. A lumbar puncture with cerebrospinal fluid analysis excluded an underlying demyelinating condition such as multiple sclerosis [Table 2]. Additional serum studies included negative antinuclear antibodies, a normal lysozyme level of 3.4 *μ*g/mL, and a negative syphilis enzyme immunoassay screen. Anticoagulation was subsequently discontinued and replaced with aspirin 81 mg daily, as the dissection was determined to be nonflow limiting, with an uncertain duration. The patient was discharged after 5 days with instructions for outpatient follow‐up with primary care and neurology. At follow‐up, she reported improvement in her symptoms, including a gradual return of visual acuity.

Figure 1Computed tomography (CT) imaging on initial presentation. (a) CT angiography (CTA) of the neck with and without contrast showing short Segment 2 dissection of the right vertebral artery at the C4 and C5 levels, 1.5 cm in length. (b) CTA of the head with and without contrast showing moderate segmental narrowing of the proximal right ophthalmic artery, whereas the artery remains patent distal to this narrowing. (c) Zoomed in view of the ophthalmic artery stenosis seen in image B.

**Figure:**
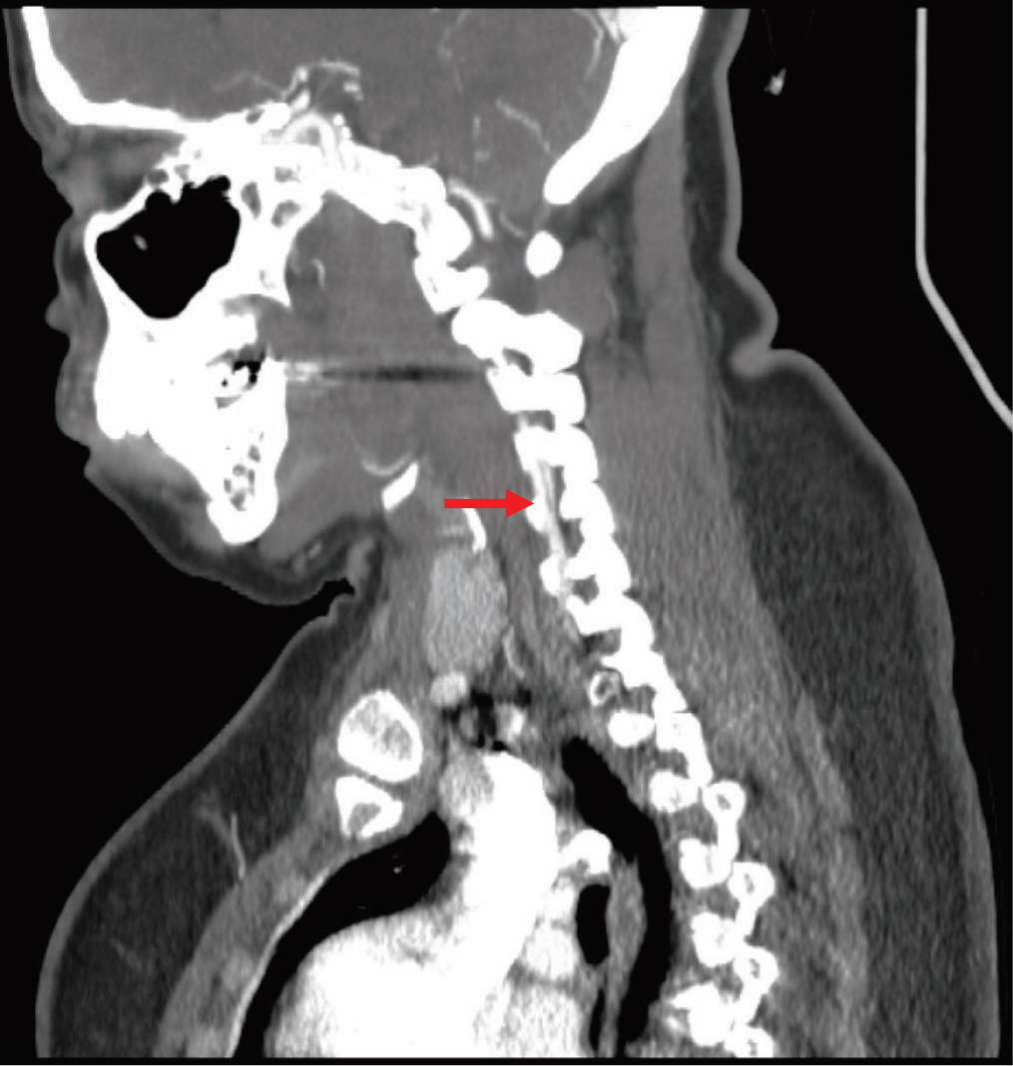
(a)

**Figure:**
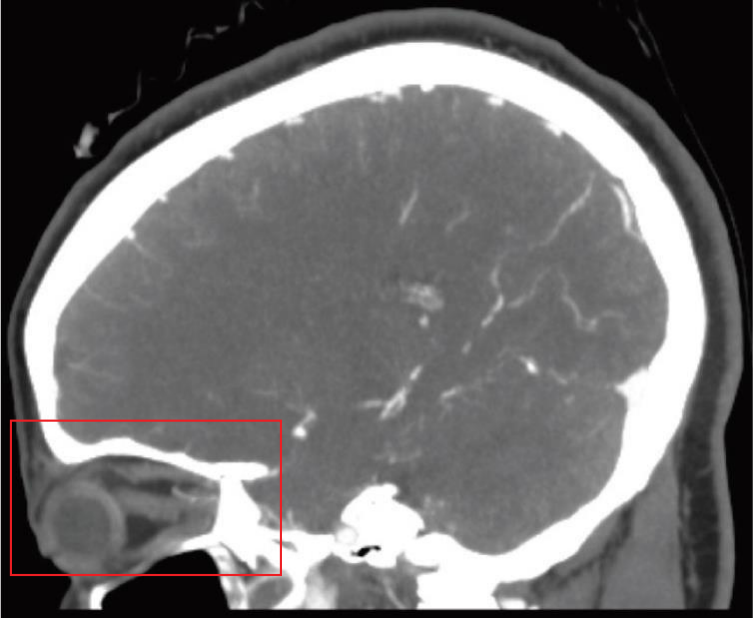
(b)

**Figure:**
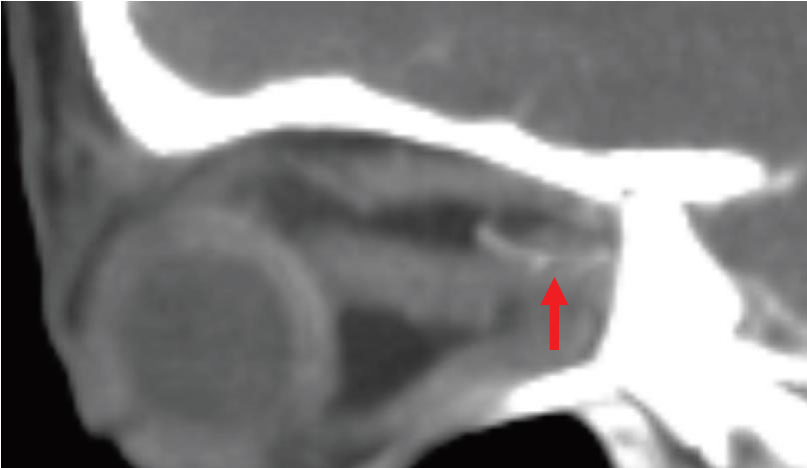
(c)

Figure 2T1‐weighted MRI with and without contrast of the orbits. (a) Slight enhancement of the right optic nerve concerning for acute optic neuritis. (b) Zoomed‐in view of the right optic nerve.

**Figure:**
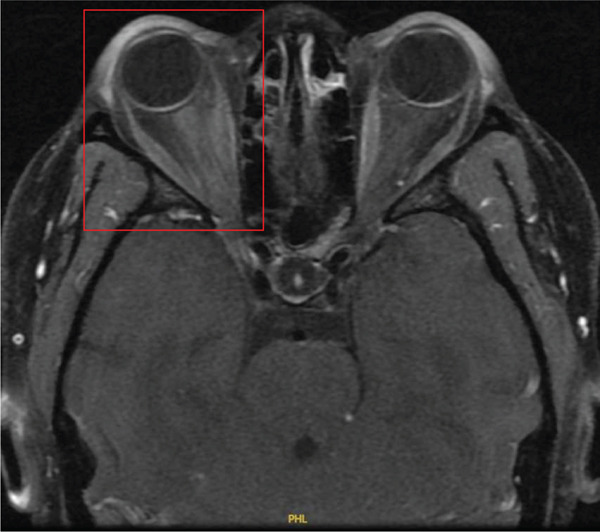
(a)

**Figure:**
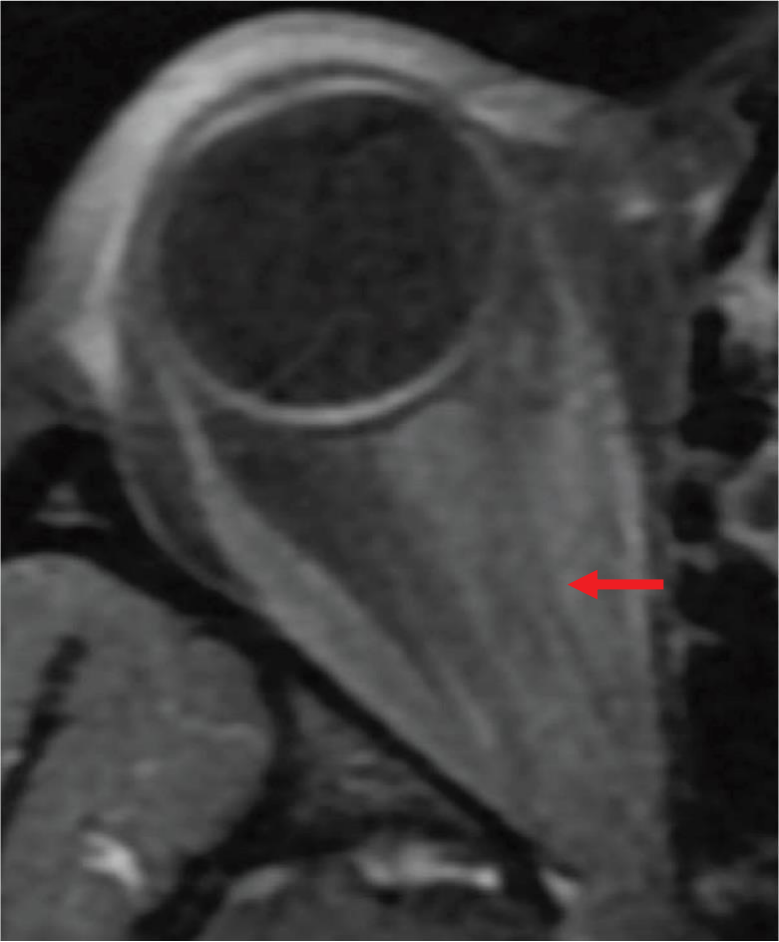
(b)

**Table 2:** CSF values acquired via lumbar puncture.

	Patient values		Normal reference values
Cerebrospinal fluid (CSF) analysis	Tube 1	Tube 4	
WBC	0.007	0.005	0.000–0.005 × 10^3^/uL
RBC	300	13	0–0 cells/uL
Mononuclear cells (count)	0.005	0.005	0.070–0.110 cells/uL
Mononuclear cells (%)	71.40%	100%	70%–100%
Polymorphonuclear (count)	0.002	0	0.000–0.006 cells/uL
Polymorphonuclear (%)	28.60%	0%	0%–6%
Glucose	81	—	45–75 mg/dL
Protein	25	—	15–45 mg/dL

Additional CSF studies			
CSF stain	No organism seen		No organism
CSF culture	No growth in 3 days		No growth
CSF protein electrophoresis	No mono‐ or oligoclonal immunoglobulin bands detected		No abnormal bands

## 3. Discussion

Spontaneous VAD is a known cause of posterior circulation stroke in younger adults, often underdiagnosed due to its varied and sometimes subtle presentation [1]. The classic presentation includes neck pain or headache with neurologic symptoms such as dizziness, visual changes, or focal deficits [2, 4, 8]. Our patient′s presentation with isolated eye pain and blurry vision made the diagnosis challenging, especially in the absence of trauma or other risk factors.

Ophthalmic artery involvement is exceedingly rare in vertebral artery dissection, with most cases of ocular ischemia more commonly associated with internal carotid artery pathology [9, 10]. Although prior case reports have described complex collateral flow patterns between the carotid and ophthalmic arteries [10], the ophthalmic artery narrowing in our patient was most likely an incidental radiographic finding rather than the cause of her optic neuritis. Golub et al. described a case involving a 49‐year‐old healthy woman who presented with bilateral internal carotid artery dissection alongside simultaneous VAD [10]. In that case, the collateral response included extensive anastomoses between the external carotid artery and internal carotid artery via the ophthalmic artery [10].

The existing literature and clinical guidelines consistently indicate that although visual disturbances can occur in vertebral artery dissection, they are typically due to occipital lobe ischemia or brainstem involvement, rather than primary retinal ischemia. Retinal ischemia occurs in approximately 50%–95% of carotid artery dissection cases, but specific incidence rates for VAD are not well documented [8]. VAD more commonly presents with symptoms such as headache, neck pain, vertigo, nausea, visual disturbances, or syncope. When visual symptoms are present, they usually reflect posterior circulation involvement rather than direct retinal ischemia [8].

In our patient, MRI demonstrated mild enlargement and contrast enhancement of the right optic nerve, findings consistent with optic neuritis. On coronal postcontrast sequences, enhancement was confined to the optic nerve substance without disproportionate enhancement of surrounding soft tissue or adjacent vascular structures. The differential diagnosis included demyelinating, inflammatory, or ischemic etiologies. Although localized vasculitis confined to the optic nerve region cannot be entirely excluded, there was no radiographic evidence of circumferential vessel wall thickening, perineural inflammatory changes, or segmental arterial wall enhancement suggestive of active vasculitis. Cerebrospinal fluid analysis did not support a demyelinating process. Notably, we were unable to identify any literature directly linking optic neuritis to ophthalmic artery stenosis. Although a causal relationship between the vascular findings and optic neuritis cannot be definitively established, the imaging and laboratory findings favored primary optic neuritis with coexisting vascular abnormalities.

The diagnosis of VAD was based on contrast‐enhanced CT angiography demonstrating focal luminal narrowing and irregularity at the C5–C6 level. Although high‐resolution MRI, vessel wall imaging, or BPAS techniques may provide additional confirmation through visualization of mural hematoma or intimal flap, these studies were not performed as CTA findings were considered sufficient for clinical management and the patient remained neurologically stable.

As of the time of this writing, the patient has not been diagnosed with any underlying autoimmune condition. The chronicity of the VAD remained uncertain. In the absence of progressive neurological deficits or imaging features suggestive of acute ischemia, the vascular abnormalities were managed conservatively and interpreted as coexisting findings rather than definitively causative of the optic nerve pathology.

This case emphasizes the importance of including cervical arterial dissection in the differential diagnosis of young patients presenting with painful vision loss. A slightly abnormal fundoscopic exam, even in the absence of neurologic findings, should prompt vascular imaging such as CTA or MRA of the head and neck.

## 4. Conclusion

This case highlights a rare presentation of spontaneous VAD with concurrent narrowing of the ophthalmic artery in a young, otherwise healthy woman. The patient presented with isolated blurry vision and headache, with minimal neurologic findings. Vascular imaging was essential in identifying both the optic nerve pathology and the coexisting vascular abnormalities.

Clinicians should consider cervical artery dissection in young patients with unexplained ocular symptoms and subtle exam findings. Early recognition and intervention may significantly alter the course of potential ischemic events.

## Funding

This research did not receive any specific grant from funding agencies in the public, commercial, or not‐for‐profit sectors. It was conducted as part of the authors′ employment at HCA Florida St. Lucie Hospital.

## Disclosure

This research was supported (in whole or in part) by HCA Healthcare and/or an HCA Healthcare affiliated entity. The views expressed in this publication represent those of the authors and do not necessarily represent the official views of HCA Healthcare or any of its affiliated entities. The employer had no role in the study design, data collection and analysis, manuscript preparation, or decision to publish.

## Consent

Written informed consent was obtained from the patient for publication of this case report and accompanying images. Documentation is available for review by the journal′s editorial office upon request.

## Conflicts of Interest

The authors declare no conflicts of interest.

## Data Availability

The data that support the findings of this study are available on request from the corresponding author. The data are not publicly available due to privacy or ethical restrictions.
